# Virginal breast hypertrophy in a 14‐year‐old girl: A case report

**DOI:** 10.1002/ccr3.3498

**Published:** 2020-11-06

**Authors:** Biraj Pokhrel, Sandesh Gautam, Samit Sharma, Nishan Babu Pokhrel, Naveen Chandra Bhatta, Sangam Rayamajhi, Jayan Man Shrestha

**Affiliations:** ^1^ Department of Plastic Surgery Tribhuvan University Institute of Medicine Kathmandu Nepal

**Keywords:** adolescent, macromastia, mammoplasty, puberty

## Abstract

Reduction mammoplasty with free nipple graft can be a good choice for early pubertal patients requiring massive reduction given its low recurrence and greater patient satisfaction.

## Graphical abstract

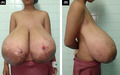

## 1 INTRODUCTION

A 14‐year‐old girl presented with rapid growth of bilateral breasts occurring within a year, causing chest discomfort and pressure sores in the inframammary fold. Bilateral reduction mammoplasty with free nipple‐areola graft was performed. Breast tissue weighing nine kilograms, accounting for 15% of total body weight, was excised.

Virginal breast hypertrophy (VBH) is a benign condition manifesting as atypical, rapid, and continued increase in either unilateral or bilateral breast size, disproportionate to other body parts during puberty.^1^, ^2^ Patients experience an initial period of quickened growth for three to six months. If left untreated, a longer period of slower growth may last for childbearing years.^3^ Reduction mammoplasty with its modifications is generally advisable to decrease the mass effect of the breast. However, there are chances of recurrences.^4^ This is an extremely uncommon condition arising sporadically, though a familial case has been reported.^5^ Hoppe et al.^6^ mentioned sixty‐five cases reported in the literature till 2010 AD. Later on, Hisham et al.^7^ acknowledged nine more cases in addition to Hoppe et al. from 2010 to 2016 AD. In developing country like Nepal, breast and cosmetic surgery is still in its growing phase and such cases barely come to notice. To the best of our knowledge, this is the first case reported from Nepal. Herein, we describe a case of a 14‐year‐old girl, who was diagnosed with this rare entity and corrected surgically.

## 2 CASE PRESENTATION

A 14‐year‐old female student from eastern Nepal visited our center with massive and gradually progressive enlargement of bilateral breasts for a year, along with chest discomfort and pressure sores in the inframammary folds and shoulders due to bra straps. She hesitated to talk about her increasing breast size with her parents and incapacitated her from attending school and social activities.

**FIGURE 1:**
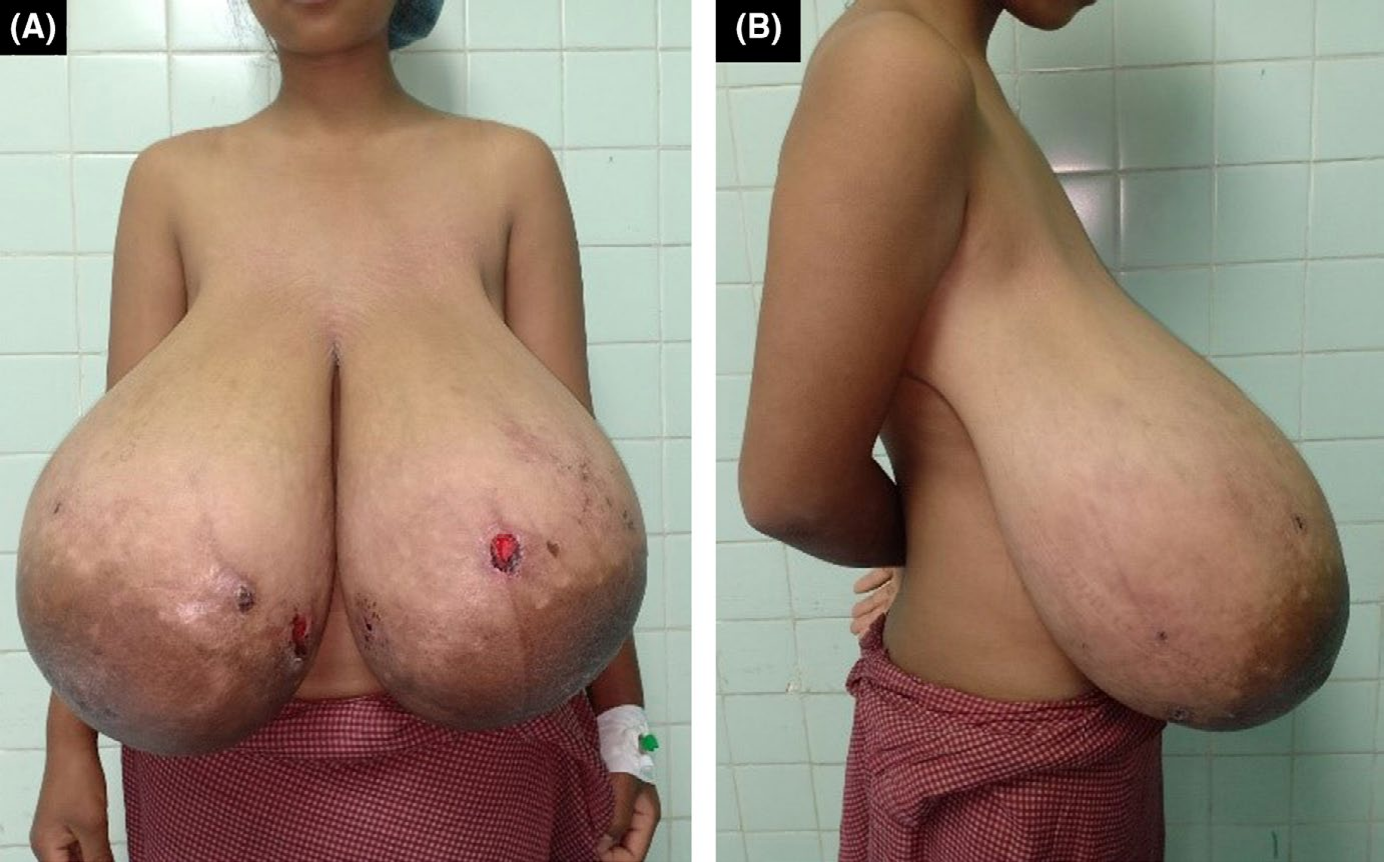
The anterior (A) and lateral (B) views of the body showing breast hypertrophy just before surgery

On examination, her bilateral breasts were pendulous and enlarged disproportionately to other body parts, with widened areola and multiple areas of pressure necrosis over the skin (Figure 1). Breasts were nontender and firm on palpation, without any discrete masses. Axillary lymph nodes were not enlarged. She had normal body mass index (BMI) of 23.15 kg/m^2^ (weight = 60 kg and height = 161 cm). Complete blood counts, C‐reactive protein, thyroid function test, follicle‐stimulating hormone, luteinizing hormone, estradiol, progesterone, and prolactin were within normal limits. No imaging investigations were done.

**FIGURE 2:**
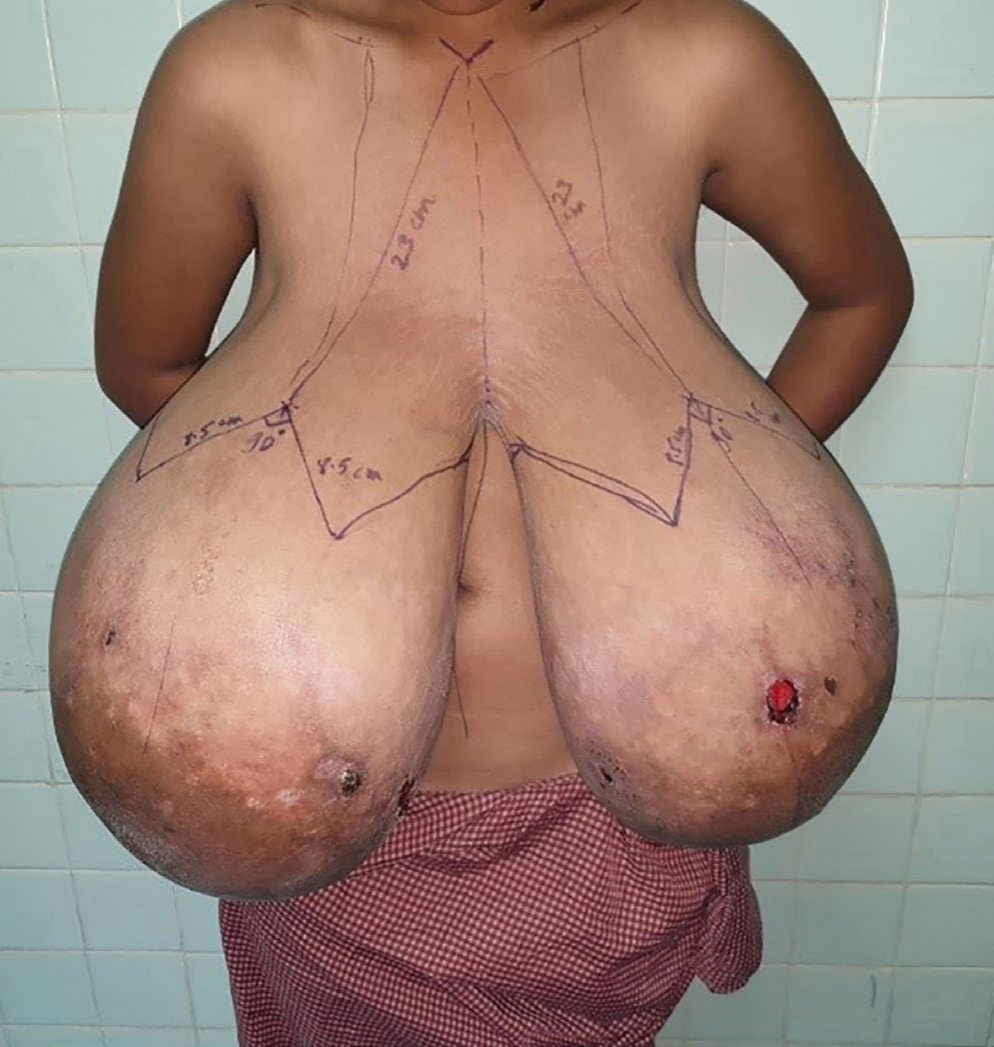
Markings of neo‐NAC on breast meridian, 23 cm from the suprasternal notch, and neo‐NAC to inframammary fold length of 8.5 cm

**FIGURE 3:**
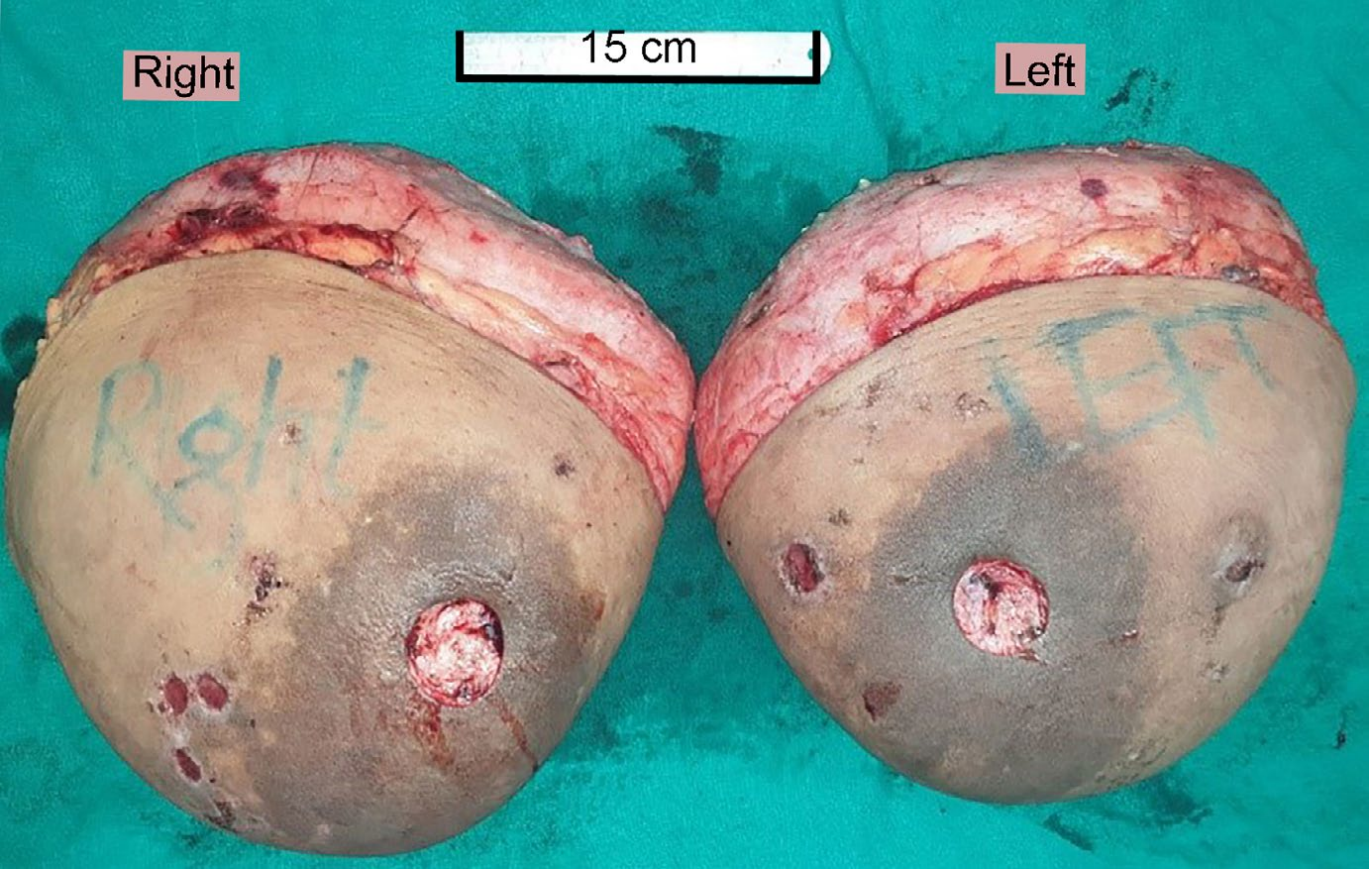
Removed breast tissues weighing five and four kgs were excised from the right and left breasts, respectively

**FIGURE 4:**
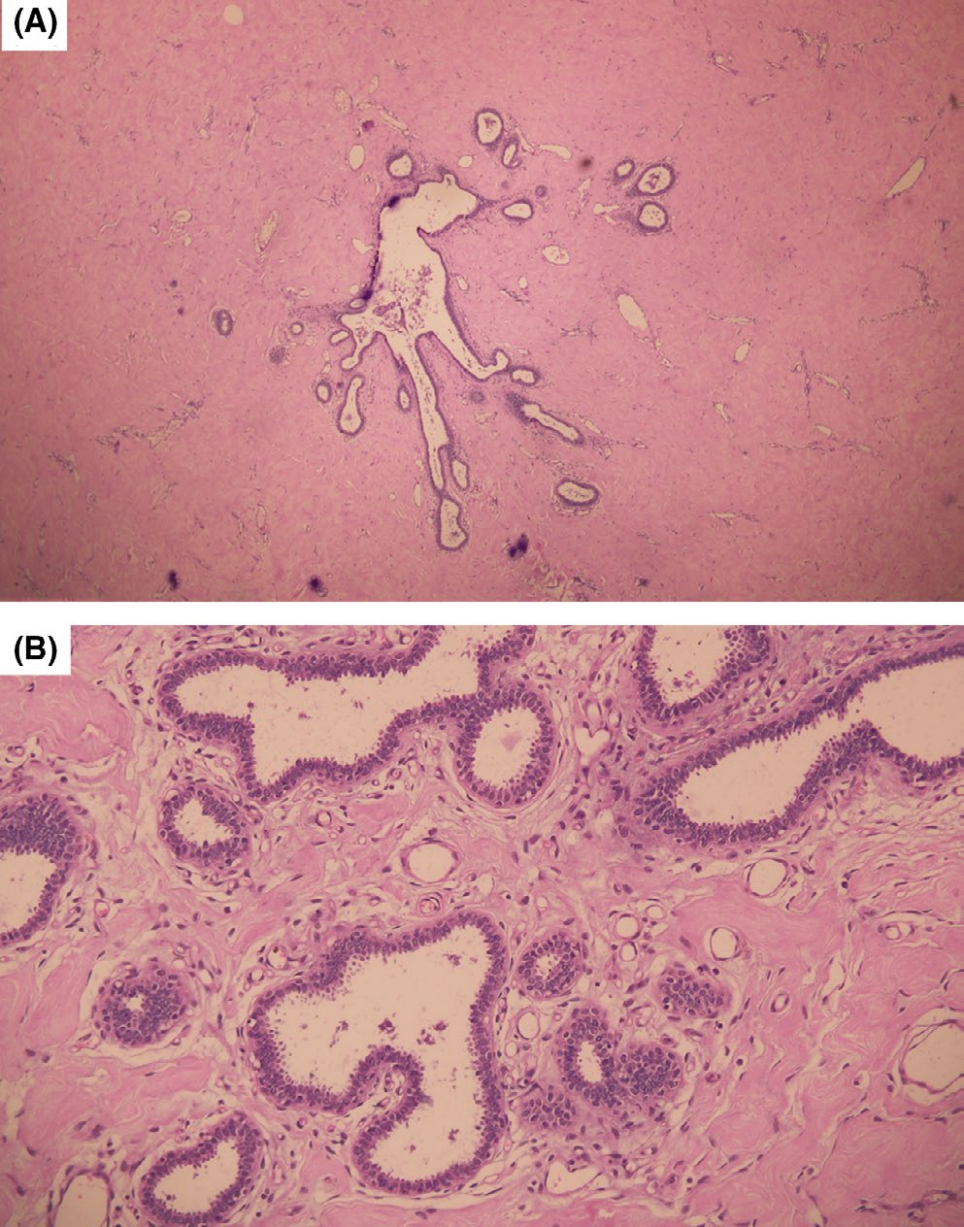
Panel A (hematoxylin and eosin, ×40), left breast specimen showing a section of the mammary duct with extensive collagen deposited areas; Panel B (hematoxylin and eosin, ×100), magnified view of mammary duct showing normal appearing inner ductal and outer myoepithelial cells without any atypia, periductal stromal fibrosis

Bilateral reduction mammoplasty with free nipple‐areola graft was performed. Markings for neo–nipple‐areolar complex (neo‐NAC) on both breasts were done at 23 cm from the suprasternal notch on breast meridian, with neo‐NAC to inframammary fold (IMF) length of 8.5 cm (Figure 2). Wise pattern skin incision was made. Full‐thickness NAC was excised bilaterally. Skin and parenchymal resection of medial and lateral wedges of tissues and whole breast tissue inferior to the neo‐NAC was excised till the fascia, which revealed encapsulated solid breast tissue. Specimens consisting of skin and breast tissue weighing five kilograms (kgs) and four kgs were excised from the right and left breasts, respectively (Figure 3). Medial and lateral flaps were brought together and hitched at IMF. The skin over the intended neo‐NAC was de‐epithelized. Then, the full‐thickness NAC was grafted over it and secured with the tie over (bolster dressing). Neo‐IMF and vertical limb of inverted T were sutured, and the excised tissue was sent for histopathology. Histopathology revealed extensive fibrosis with collagen deposition in the mammary ducts lined by inner ductal epithelial cells and outer myoepithelial cells (Figure 4). There was focal cellular stroma without atypia. This was suggestive of pubertal macromastia without evidence of malignancy.

**FIGURE 5:**
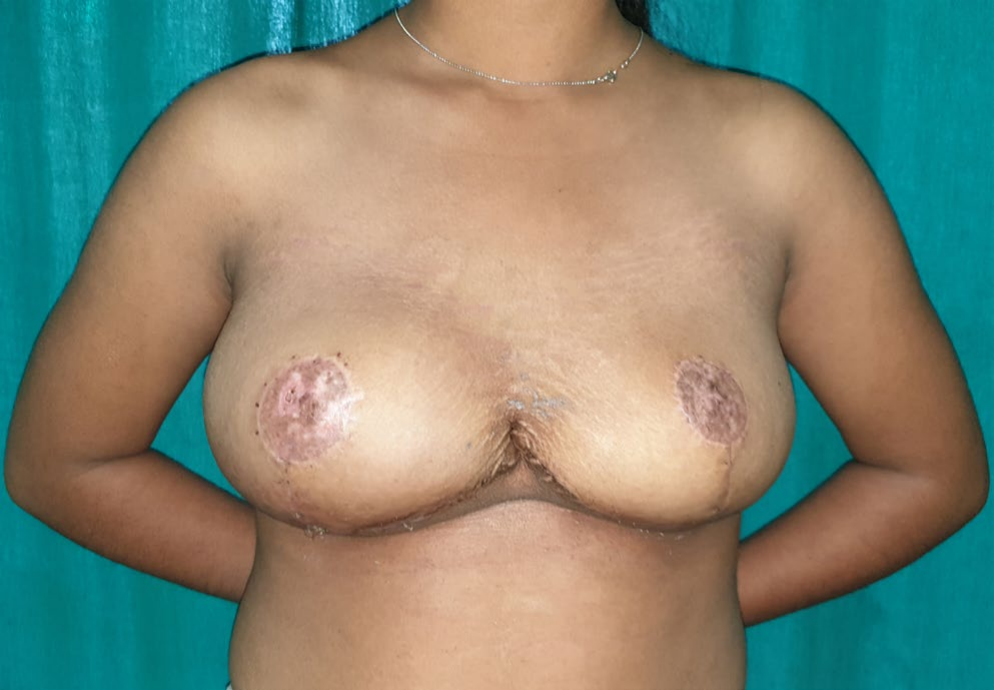
Condition of breasts after one month of surgery

Her chest discomfort, and neck and back pain subsided (Figure 5). She was doing fine and was satisfied with the outcome of the surgery. She improved her social interactions as well. There was no recurrence after eleven months of follow‐up.

## 3 DISCUSSION

Breast remains largely unchanged from birth to puberty though the lobular‐alveolar, and the ductal systems grow under the strong influence of estrogen and progesterone.^8^ From puberty onwards, normal breast development occurs over 2‐5 years involving all tissues of the breast due to the result of the anterior pituitary hormone through the follicle‐stimulating hormone, luteinizing hormone, growth hormone, and adrenocorticotrophic hormone along with the estrogen and progesterone. Estrogen, progesterone, and prolactin have the strongest effect on ductal growth and lobular‐alveolar development.^8^, ^9^, ^10^ However, sometimes massive or excessive growth of the unilateral or bilateral breasts can occur despite the normal level of gonadal hormones. Though the exact cause remains unknown, it is believed to be due to idiopathic end‐organ hypersensitivity to the normal level of gonadal hormones.^11^ Molecular studies regarding the VBH show that *PTEN* gene might have a role in its pathogenesis.^12^

With the increasing size and weight of the breast, it looks disproportionately large and pendulous. It may cause breast tenderness, postural problems, respiratory problems, and neck and back pain.^5^, ^9^, ^13^ Due to chronic irritation of the bra straps, shoulders can be grooved and scarred.^5^, ^9^ Similarly, intertrigo and stretch marks can also be observed.^13^

In Nepal, adolescent girls are reluctant to talk about issues relating to the breast, which might be influenced by the prevailing culture. With the rapid growth of breast causing sudden change of the body shape, in comparison with peers, and unfitting clothes may lead to social embarrassment, loss of social life, confidence, and depression.^3^ In our case, the patient had limited her social activities and stopped going to the school due to the embarrassing size of her breasts.

Diagnosis is straightforward, but wide differentials like fibroepithelial tumors (fibroadenoma and phyllodes tumor) and fibrocystic changes, which are more common than VBH in this age group, should be ruled out.^1^, ^14^

Management includes either surgical or medical method or both. To arrest the breast growth before or after surgery, medical modulators like tamoxifen citrate and medroxyprogesterone have been used. But their results are variable, and side effects cannot be overlooked.^3^, ^15^

Surgery is the best treatment modality, which can be considered to eliminate the physical symptoms. Subcutaneous mastectomy with implants and reduction mammoplasty with its modifications, free nipple graft, or pedicle‐based technique are the available options.^6^ Though subcutaneous mastectomy with implants has the least chance of recurrences, it is not the first‐line choice for many.^3^, ^6^ Implants have the lifelong risk of complications. Also, in the developing country like Nepal such costly operation is not feasible and expensive implants cannot be afforded by the patient.

Reduction mammoplasty with free nipple graft is the most common preferred approach. Patients who need large volume reduction (resected tissue greater than two kgs on each side or sternal notch to the nipple distance greater than 40 cm), as in our case, are generally advocated for it.^16^ Fiumara et al.^4^ accentuated the statistical evidence of decreased chance of hypertrophic recurrence with the use of free nipple graft than pedicle‐based technique (*P* = .005). Hisham et al.^7^ reported a case of massive reduction surgically corrected with reduction mammoplasty with free nipple graft without recurrence in five year follow‐up. Our case, with no recurrence till date, also further supports this evidence that free nipple graft reduction mammoplasty can be the choice in the patient requiring massive reduction. On the contrary, the pedicle‐based technique has a greater chance of recurrences needing additional surgery. It utilizes a long fold of pedicle and can also compromise the blood supply during large volume resection and lead to increased risk of nipple.^16^, ^17^ Also, it has an increased rate of complications.^17^

However, free nipple grafting has certain disadvantages. It results in the loss of lactation, variable return of sensation and contractility of the nipple, graft failure, and at times pigmentary changes of the nipple‐areolar complex.^16^, ^18^, ^19^ It is necessary to counsel the patient and her family members about the limitations of this procedure.

## 4 CONCLUSION

Breast and cosmetic surgery is in its infancy stage in Nepal, and the VBH is an uncommon presentation. Reduction mammoplasty with the free nipple graft alone can be a first‐line surgical option for the early pubertal patients with voluminous breasts.

## CONFLICT OF INTEREST

The authors declare that there is no conflict of interest regarding the publication of this paper.

## AUTHOR CONTRIBUTIONS

BP and SG: involved in conceptualization, collecting information, and manuscript writing. NBP, SS, and NB: participated in the literature review and edited the draft. JS, SR, and SS: involved in patient care team and also independently reviewed the manuscript. BP, SG, NBP, SS, and NB: re‐edited the draft and reshaped it into this manuscript. All authors: approved the final version of the manuscript and agreed to be accountable for all aspects of the work in ensuring that questions related to the accuracy or integrity of any part of the work are appropriately investigated and resolved.

## ETHICAL APPROVAL

Need for ethical approval waived. Consent from the patient and her father deemed to be enough.

## CONSENT FOR PUBLICATION

Written informed consent was obtained from the patient and her father for publication of this case report and any accompanying images. A copy of the written consent is available for review by the editor‐in‐chief of this journal.

## Data Availability

Not applicable.
